# Citrinin exposure affects oocyte maturation and embryo development by inducing oxidative stress-mediated apoptosis

**DOI:** 10.18632/oncotarget.15776

**Published:** 2017-02-28

**Authors:** Yu Wu, Na Zhang, Ying-Hua Li, Liang Zhao, Mo Yang, Yimei Jin, Yong-Nan Xu, Hongyan Guo

**Affiliations:** ^1^ Department of Obstetrics and Gynecology, Peking University Third Hospital, Beijing 100191, P.R. China; ^2^ College of Agriculture, Yanbian University, Yanji 133002, P.R. China; ^3^ Department of Obstetrics and Gynecology, Beijing Jishuitan Hospital, Beijing 100035, P.R. China

**Keywords:** citrinin, oocyte, cytoskeleton, apoptosis, oxidative stress

## Abstract

Citrinin is one of the mycotoxins and has been shown to have various toxic effects in animals and humans. Although previous study showed the toxic effects of citrinin on the female reproductive system, especially on oocyte maturation, however, the causes or mechanism of citrinin on oocyte quality is unclear. In present study we deeply investigated this topic. We found thatcitrinin toxin exposure inhibited mouse oocyte maturation and early embryo development. Further investigation showed that the actin distribution in oocytes and embryos was disrupted, and the reduced expression of actin nucleator ARP2 expression in the oocyte cortex further confirmed this. We also found that meiotic spindle morphology was abnormal after citrinin treatment. These results indicated that citrinin toxin exposure could disrupt cytoskeleton dynamics to affect oocyte maturation and early embryo development. We also examined the ROS level and early apoptosis marker Annexin signals, and the results showed that both levels increased, indicating that citrinin induced oxidative stress and further resulted in oocyte early apoptosis. Taken together, our results indicated that citrinin toxin exposure could reduce mouse oocyte maturation and early embryo development capability by affecting cytoskeletal dynamics, which may be due to the oxidative stress induced early apoptosis.

## INTRODUCTION

Citrinin toxin is one of the mycotoxins that are secondary metabolite produced by fungi, such as Penicillium citrinum, the Monascus species and the Aspergillus species. Citrinin is generally found from the bad stored crops, feeds or foods. And citrinin, similar with other myocotoxins, was shown to have multiple toxic effects on different organs, especially in reproductive system [1]. Besides citrinin, the mycotoxin family also includes Zearalenone, Aflatoxin, Deoxynivalenol, Ochratoxin et al. These mycotoxins were all show to be toxic to the mammalian oocyte maturation and embryo development in mouse and pig [2–5]. Critrinin toxin was also reported to affect mouse oocyte maturation and embryo development [6, 7]. However, the possible causes or mechanisms were still unclear. In this study, we tried to explore this scientific question from the cytoskeleton dynamics and oxidative stress-induced apoptosis aspects.

Oocyte maturation and early embryo development depended on cytoskeleton dynamics. After germinal vesicle break down (GVBD), microtubule assembled to the meiotic spindle in oocytes, and leaded the chromosome congression. Actin filaments were the main power to push the meiotic spindle to the cortex of the oocytes to ensure the asymmetric cell division, which generated the polar body to retain most maternal components in the egg for the early embryo development. After the spindle movement, actin filaments formed the contractile ring to initiate the cytokinesis, and the first meiosis (meiosis I) completed after the polar body extrusion [8]. Similar cellular process occurred in the early embryo development except the spindle movement. During these processes, lots of molecules involved into this proess, for example, actin nucleator ARP2 regulated actin assembly in mouse oocyte to ensure the oocyte asymmetric division [9], and ARP2 also involved into embryo development [10]. Therefore, cytoskeleton dynamics are critical for oocyte meiosis and embryo cleavage.

Oxidative stress and apoptosis were all have reverse effects on oocyte maturation and early embryo development. Generally oxidative stress indicated the imbalance between the systemic generation of reactive oxygen species (ROS) and the capability of the biological system to detoxify the reactive intermediates or to repair the existed damage. In the reproductive system, ROS are mainly generated within the follicle, and the ovulation process also produced ROS. Several studies showed that the mycotoxins ZEN and T-2 toxin generated oxidative stress had toxic effects on mammalian oocyte quality [4, 11], while the melatonin could protect oocytes from oxidative stress [12]. While in human cervical cancer cells, oxidative stress was shown to be the main cause for the T-2 toxin-mediated toxicity [13]. For apoptosis, the mycotoxin members like AFB1, ZEN and DON were all reported to be related with apoptosis in mammalian oocytes. Exposure to AFB1, ZEN or DON were all caused the increased early apoptosis level in mouse and pig oocytes [3, 5, 11]. T-2 toxin was also shown to induce mammalian somatic cells apoptosis. Previous study showed that in U937 cells caspase-2 was necessary for T-2 toxin-induced apoptosis, and that apoptotic signals were mainly moved via caspase-8 and caspase-3 instead of the mitochondrial pathway [14].

Lots of the identified aneuploidies in embryos were generated from meiotic processes errors in mammalian oocytes [15]. Due to the facts that citrinin toxin was shown to have the toxic effects on the reproductive systems of mammals, especially in mouse oocyte and embryos, in present study, we investigated the toxic effects of citrinin on spindle formation and actin distribution during oocyte and early embryo development. More importantly, we also examined the ROS level and early apoptosis signal Annexin to give the explanation for the adverse effects of citrinin toxin on oocyte and embryo quality. Our findings will provide the possible causes or the mechanism of the toxic effects of citrinin toxin on oocyte and embryos from subcellular structure and cellular process aspects.

## RESULTS

### Citrinin toxin exposure affects mouse oocyte maturation and early embryo development

**Figure 1:**
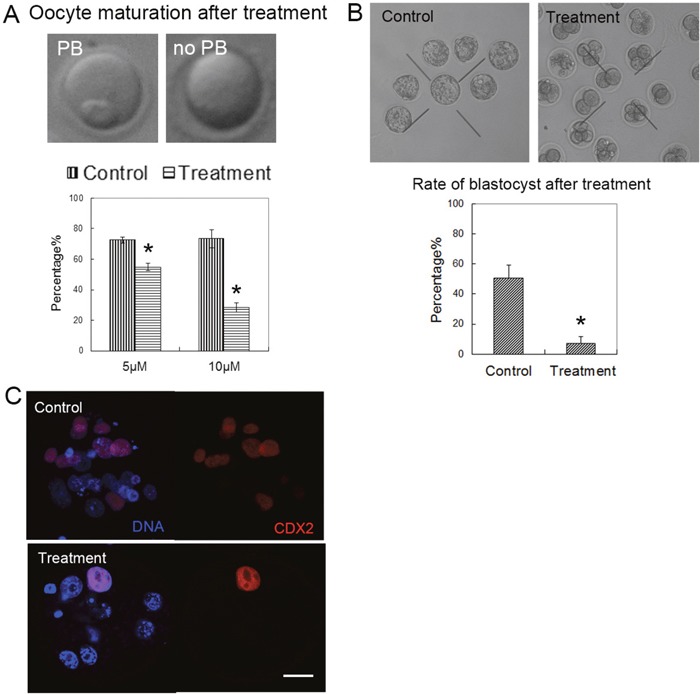
Citrinin toxin exposure affected oocyte maturation and early embryo development **(A)** The oocytes were cultured with 5 μM, 10 μM citrinin toxin for 12 h. Most oocytes developed to the MII stage in the control group, however, most of oocytes failed to reach to the MII stage. **(B)** The blastocyst embryo rate was significantly reduced with citrinin toxin treatment. *, significantly different (p < 0.01). **(C)** The expression of CDX2 in the embryos. In the control embryo, CDX2 expressed in the TE cells; while in the treated embryo, there are barely CDX2 signal. CDX2, red; DNA, blue. Bar = 20 μm.

To confirm the toxic effects of citrinin on oocyte and embryos, we used the polar body extrusion and blastocyst formation rate as the index for the examination of the effects of citrinin toxin exposure. We captured the sample oocytes from mouse ovaries and cultured for 12 h with 5 μM and 10 μM citrinin toxin. As shown in Figure 1A, we found that the polar body extrusion rate of mouse oocytes was significantly reduced after exposure to citrinin toxin (72.6 ± 2.0%, n=57 versus 54.9 ± 2.3%, n=84 and 73.4 ± 5.9%, n=66 versus 28.4 ± 3.3%, n=72; p < 0.05). And we chose 10 μM citrinin exposure as the standard treatment concentration in our present study. We then examined the blastocyst formation, and the results showed that after citrinin treatment, most embryos could not develop to the blastocyst stage (50.8 ± 8.5%, n=64 vs 7.3 ± 4.5%, n=86, p<0.05, Figure 1B). To further confirm this results, we stained the CDX2 antibody. And the results showed that the signal of the TE cell marker protein CDX2 was abnormal in the treatment group. In the control blastocyst, CDX2 localized to TE cells, while there was no clear fluorescence signal in the citrinin treatment embryos, indicating that the treated-embryos did not develop to the blastocyst stage, which further confirm our results above (Figure 1C).

### Citrinin exposure reduces actin filaments distribution in both oocytes and embryos

**Figure 2:**
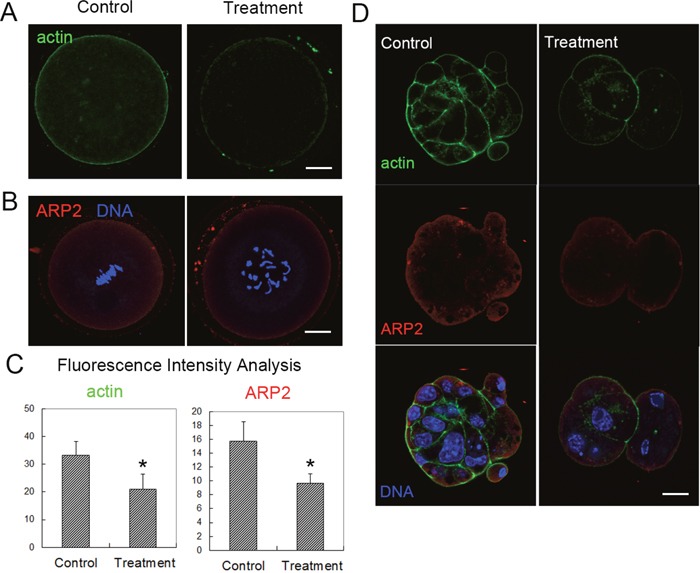
Citrinin toxin exposure affected actin filament dynamics in mouse oocytes and embryos **(A)** Actin distribution was disrupted after citrinin toxin exposure. Actin, green. **(B)** ARP2 fluorescence intensities were reduced in citrinin toxin treated oocytes. ARP2, red; DNA, blue. Bar = 20 μm. **(C)** Average actin and ARP2 fluorescence intensity analysis in mouse oocytes. *, significantly different (p < 0.05). **(D)** Actin distribution and ARP2 was disrupted after citrinin toxin exposure in mouse embryos. Actin, green; ARP2, red; DNA, blue. Bar = 20 μm.

Our results above confirmed that citrinin had toxic effects on mouse oocyte maturation and early embryo development. We then tried to study the possible causes for the reduced developmental competence of oocytes and embryos after citrinin toxin treatment. We first examined the distribution of actin filaments. We cultured the oocytes for 8 h, and we found that in these metaphase I (MI) oocytes after citrinin exposure, the fluorescence signals at the membrane were much weaker than those in control MI oocytes (Figure 2A). To further confirm this, we also examined the fluorescence signal of ARP2, an actin nucleator which belong to Arp2/3 complex and regulates actin filaments assembly, and we found that the expression of ARP2 was also much weaker than the control group (Figure 2B). We also did fluorescence intensity analysis for actin and ARP2, and the results further proved our findings (For actin, 33.3 ± 5.0 versus 21.2 ± 5.4, n=30, 10 for each repeat; p < 0.05; for ARP2, 15.7 ± 2.9 versus 9.7 ± 1.3, n=30, 10 for each repeat, Figure 2C). We then examined the actin filaments in mouse embryos after citrinin treatment, and the similar results were found. In the control embryos, actin filaments and ARP2 showed strong fluorescence signals, while in the citrinin exposure group, the signals were much weaker in these treated embryos (Figure 2D). These results indicated that actin filaments distribution was disturbed after citrinin treatment.

### Citrinin exposure causes the spindle formation defects in oocytes

**Figure 3:**
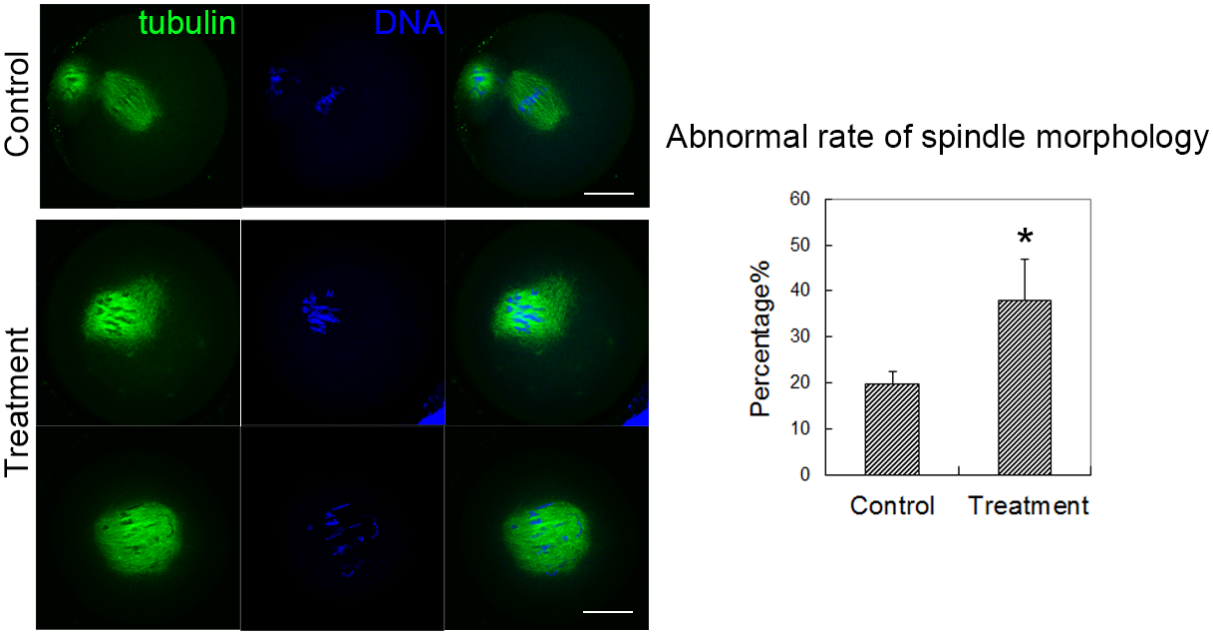
Citrinin toxin exposure affected spindle formation in mouse oocytes In the control group, the oocyte spindle exhibited normal morphology; while in the treatment group, the oocyte spindle showed aberrant spindle with mis-aligned chromosomes. The analysis data showed that the abnormal rate of spindle morphology increased significantly after citrinin toxin treatment, p<0.05.

We next examined the spindle formation in mouse oocytes after citrinin treatment, since spindle was one important sub-cellular structure for the chromosomes segregation during oocyte meiotic maturation. We cultured the oocytes for 12 h, and we stained the tubulin antibody to observe the morphology of the spindle. In the control group, the sample oocytes reached metaphase II (MII) stage and the spindles showed normal morphology. While in the treatment group, the sample oocyte spindles showed aberrant morphology, and the chromosome alignment was disrupted (Figure 3). We also analyzed the abnormal rate, and the statistical data showed that the proportion of abnormal spindles in the citrinin exposure group was significantly higher than that in the control group (19.7 ± 2.9%, n=85 versus 38.0 ± 8.9%, n=66; p < 0.05; Figure 3). Therefore, these results above suggested that citrinin toxin exposure caused aberrant meiotic spindle formation.

### Citrinin exposure causes oxidative stress induced early apoptosis in oocytes

**Figure 4:**
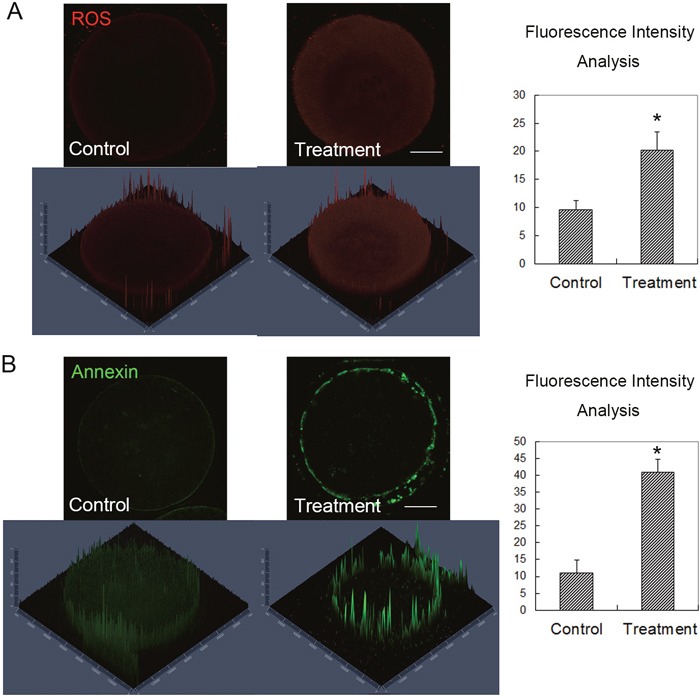
Citrinin toxin exposure induced oxidative stress and early apoptosis in mouse oocytes **(A)** After citrinin toxin treatment, the ROS level was significantly increased in the treated oocytes, while the average ROS fluorescence intensity analysis showed that ROS signal increased significantly in the treated mouse oocytes. *, significantly different (p < 0.05), Bar = 20 μm. **(B)** After citrinin toxin treatment, the Annexin signal, which presented the early apoptosis level was significantly increased in the citrinin treated oocytes, while the average Annexin fluorescence intensity analysis showed that Annexin signal increased significantly in the treated mouse oocytes. *, significantly different (p < 0.05), Bar = 20 μm.

Our results now showed that citrinin could disrupt spindle formation and actin filaments distribution, and then affect oocyte meiotic maturation and early embryo development. We then tried to understand the causes or the mechanism of the toxic effects of citrinin toxin on these cellular processes. We next examined the ROS levels in mouse oocytes. As shown in Figure 4A, ROS fluorescent signal in citrinin treated oocytes was much stronger compared to the oocytes of control group. Fluorescence intensity analysis results also confirmed this (9.6 ± 1.6 versus 20.2 ± 3.3, n=30, 10 for each repeat; p < 0.05; Figure 4A). This suggested that citrinin toxin exposure induced oxidative stress in mouse oocytes.

We next examined whether citrinin toxin exposure could induced early apoptosis, since oxidative stress always could cause the apoptosis. We stained with Annexin and the results showed that after citrinin toxin exposure, the fluorescence signal of Annexin was much stronger than that of the control sample oocytes, this indicated that early apoptotic signals increased in citrinin toxin treated oocytes (Figure 4B). We also analyzed the fluorescence intensity of Annexin, and the quantitative results showed that in the citrinin treated oocytes the Annexin signals were significantly increased compared with the control group (11.1 ± 3.8 versus 41.0 ± 3.8, n=30, 10 for each repeat; p < 0.05; Figure 4B). This results suggested that citrinin exposure to mouse oocytes could cause the oxidative stress induced early apoptosis.

## DISCUSSION

In present study, we studied the possible causes for the low developmental competence of mouse oocyte meiotic maturation and embryo development with citrinin toxin exposure. We examined the actin filaments distribution, meiotic spindle morphology, early apoptosis signal and oxidative stress in mouse oocytes after exposure to citrinin toxin. We found that citrinin toxin had toxic effects on actin expression and meiotic spindle morphology, which resulted in the failure of oocyte maturation and early embryo development, while the oxidative stress induced early apoptosis might be the reason for this.

Mycotoxins are widely existed in the feeds and were reported to have various toxic effects on animals and humans. Many studies showed that mycotoxins, including ZEN, DON and T-2 toxin could affect reproductive systems [16, 17]. For example, ZEN (zearalenone) and T-2 toxin affected the mouse, pig and zebrafish oocyte maturation and early embryo development [2, 18, 19]. Similarly, previous studies showed that citrinin toxin could affect mouse and zebrafish oocyte maturation and embryo development [6, 7, 20], however, the possible causes or mechanisms are still unclear. In present study we first examined and confirmed the toxic effects of citrinin on mouse oocyte and early embryo development, and then the main focus was that we tried to find the possible reasons.

We first examined the cytoskeleton dynamics to find out the potential mechanisms underlying the low oocyte maturation rate after citrinin exposure, which included the microtubule-formed meiotic spindle, and the actin filaments. We investigated the expressions and localization of actin filaments and spindle morphology. Microtubules formed the meiotic spindle to lead chromosome segregation while actin filaments were the main power for meiotic spindle movements and regulated cytokinesis for oocyte polar body emission. We found that citrinin toxin exposure reduced the distribution of actin filaments in both mouse oocytes and embryos. Moreover, the expression of actin nucleation ARP2, a key molecule which was shown to be involved into mouse oocyte maturation was also reduced [9], which could confirm that citrinin affected actin filament distribution in mouse oocytes. We also found that citrinin toxin exposure disrupted spindle formation, the spindle morphology was abnormal after citrinin treatment. Our results were similar to those in previous studies that showed that ZEN, DON and T-2 toxin reduced the developmental competence of mouse oocytes by disrupting the distribution and localizations of actin filaments and microtubules [4, 5, 11]. Beside mouse oocytes, several studies also showed that in pig oocytes, the toxic effects of ZEN and DON were also on cytoskeleton dynamics [21, 22]. These results together showed that not only citrinin, most mycotoxins could affect mammalian oocyte cytoskeleton dynamics, which provided us a reason for the defects of polar body extrusion from subcellular structure aspect in oocytes and embryos.

We then tried to understand which cellular process was affected to cause the subcellular structure disruption under the exposure of citrinin toxin. We first examined the oxidative stress level, and we found that ROS increased. Due the fact that oxidative stress is closely related with early apoptosis, we also stained with Annexin, the early apoptosis marker. And the results showed that the oocytes exposure to citrinin toxin suffered the early apoptosis. Citrinin was shown to affect ROS level in different models, such as fission yeast [23], HepG2 cell [24] and mouse skin [25]. While besides citrinin, the other mycotoxins, like ZEN, HT-2 toxin were all reported to cause the oxidative stress, which was indicated as ROS level [4, 11]. Several previous studies showed that many members of mycotoxins could induce apoptosis. For example, T-2 toxin treatment caused apoptosis in several cell types [26–28]. And in rats, T-2 toxin induced apoptosis in the differentiated murine embryonic stem cells and ovarian granulosa cells [29, 30], which was proved by the increased oxidative stress and apoptosis related genes expression [31]. Similar results were found about HT-2 in mouse oocytes [11]. And for the other mycotoxins, ZEN, DON and AFB1 were all reported to induce the apoptosis in mouse and pig oocytes [3–5, 32]. In mammalian oocytes, oxygen species (OS) perturbation could affected oocyte maturation [33]. Therefore, our results suggested that citrinin toxin induced oxidative stress and caused the early apoptosis, which then further disrupted mouse oocyte meiotic maturation.

In all, our results suggested that citrinin toxin exposure might induced oxidative stress-mediated early apoptosis and caused the cytoskeleton dynamics defect in mouse oocyte and embryos. These alterations may be reasons for the reduced oocyte developmental competence with the citrinin toxin exposure.

## MATERIALS AND METHODS

### Antibodies and chemicals

Rabbit polyclonal anti-CDX2 and ARP2 antibody was purchased from Sant Cruz (Santa Cruz). Mouse monoclonal anti-α-tubulin-FITC antibody, Hoechst 33258, and Phalloidin-FITC were bought from Sigma (St Louis, MO, USA). Citrinin toxin was from J&K Chemical Ltd. (Shanghai, China). Dihydroethidium was from the Beyotime Institute of Biotechnology (Nantong, China). Annexin V-FITC/EGFP Apoptosis Detection Kits were from Vazyme Biotech Co., Ltd. (Nangjing, China).

### Ethic statement

All animal experiments were conducted in accordance with the guidelines of the Animal Research Committee of Peking University Third Hospital, China. Kunming female mice (Beijing Weitonglihua Experimental Animal Center) were used (3-6 months). Mice were housed in a temperature-controlled room with appropriate dark-light cycles, fed a regular diet, and maintained under the care of the Experimental Animal Center, Peking University Third Hospital, China. The mice were sacrificed by cervical dislocation. This study was approved by the Committee of Animal Research Institute, Peking University Third Hospital.

### Citrinin treatment and oocyte/embryo culture

Germinal vesicle stage oocytes were harvested from mouse ovaries. Citrinin toxin was dissolved in dimethyl sulfoxide (DMSO) for storage. For the treatment experiments, the GV oocytes were cultured in 5-10 μM Cirinin toxin in M16 medium (Sigma) at 37°C in a humidified atmosphere with 5% CO_2_. The concentration of citrinin toxin was adopted according to the previous study [20]. The control oocytes were cultured in M16 with the same concentration of DMSO. After culture for different time, the oocytes were collected for the experiments. For embryo collection and culture, we injected PMSG for 48 hours, and then the female mice were injected with hCG and mated with male mice. Zygotes were collected after 16 h and cultured in KSOM medium (Chemicon) under paraffin oil at 37 degree and 5% CO2. Embryos were collected for immunostaining after different times in culture.

### Immunofluorescence staining

After collection, the sample oocytes were fixed with 4% paraformaldehyde in phosphate-buffered saline (PBS) at room temperature for 1 h, and then the oocytes were moved to the membrane permeabilization solution (0.5% Triton X-100) for 20 min. After the incubation for 1 hour in PBS with 1% BSA at room temperature for 1 h, the oocytes were incubated with the first antibody (tubulin-FITC, CDX2 and ARP2) for 4 h. We washed the sample oocytes with phosphate-buffered saline (PBS) with 1% BSA, and then the sample oocytes were moved to the secondary antibodies for 1 h. Oocytes were stained with Hoechst 33258 for 15 min. And the samples were mounted on glass slides and was scanned with confocal laser-scanning microscope (Zeiss LSM 780 META; Jena, Germany). At least 30 oocytes were examined for each repeat.

### Early apoptosis examination by Annexin-V staining

We perform this part of experiment following the examination kit instructions of an Annexin-V staining kit (Beyotime Institute of Biotechnology, Hangzhou, China). After washing twice in PBS, the sample oocytes were stained for 10 min avoiding the light, with 100ml of binding buffer containing 5 ml of Annexin-V-FITC. Fluorescent signals were examined by the confocal microscope Zeiss LSM 780 META.

### ROS generation examination

We used the oxidation-sensitive fluorescent probe [dichlorofluorescein (DCFH)] to examine the intracellular ROS production levels, the sample oocytes were incubated with this probe for 30 min at 37 degree in D-PBS that contained 10μM DCFH diacetate (DCFH-DA) (Beyotime Institute of Biotechnology, China). After washing three times in D-PBS with 0.1% BSA, the sample oocytes were moved on glass slides. The florescence signal examination were performed with Zeiss LSM 7080 META confocal microscopy. And all the samples were scanned with the same scanning settings.

### Fluorescence intensity analysis

For the analysis of the fluorescence intensity of actin, ARP2, ROS and Annexin signals. All the samples of control and treated oocytes were mounted on the one same glass slide. Image J software was adopted to measure the region of interest (ROI), and the average fluorescence intensity for every unit area within the ROI was measured. Independent measurements using identically sized ROIs were taken for the cell membrane or the cytoplasm. The average values of all measurements were used to calculate the final average intensities between control and treated oocytes.

### Statistical analysis

For each experiment group, at least three repeats were done with the data expressed as means ± SEMs. Statistical analysis were performed by independent-sample t-tests. A p-value of <0.05 was considered significant.
